# Therapeutic mild hypothermia after cardiac arrest in shockable and nonshockable rhythms: does it improve both survival and neurological outcome?

**DOI:** 10.1186/cc12251

**Published:** 2013-03-19

**Authors:** A Gupta

**Affiliations:** 1 Fortis Escorts Heart Institute, New Delhi, India

## Introduction

Although therapeutic mild hypothermia (TMH) after resuscitation from cardiac arrest (CA) has been postulated and studied to be associated with good outcome of the patients, there is no dearth of data that does not favour TMH. Our aim was to find out whether TMH is associated with good outcome after CA in shockable rhythm (SR) compared with nonshockable rhythm (NR), in terms of survival as well as neurological outcome.

## Methods

We reviewed medical records of all CA patients (in-hospital or out-of-hospital arrest) in whom cardiopulmonary resuscitation (CPR) was performed at our hospital from 1 February 2011 to 31 January 2012 (12 months). The following information was collected: first documented rhythm, whether TMH done or not, and two outcome measures including survival to hospital discharge and neurological outcome at the time of hospital discharge. A measure of good neurological outcome was Cerebral Performance Category score 1 or 2 (CPC, five-point scale; 1 = good cerebral performance to 5 = brain death). Then we quantified the association of TMH with SR as well as NR for both the parameters of outcome - that is, survival to hospital discharge and good neurological outcome - by logistic regression analysis.

## Results

We had 297 CA patients (168 SR, 129 NR) in whom CPR was done. Return of spontaneous circulation was achieved in 90 patients. TMH was induced in 57 patients (33 SR, 24 NR). Survival to hospital discharge was observed in 27 patients (18/33 (54.5%) SR, 9/24 (37.5%) NR), out of which 18 patients (10/33 (30%) SR, 8/24 (33%) NR) had good neurological outcome. On analysis, TMH was found to be associated with increased odds of survival to hospital discharge (although statistically not significant) in SR patients compared with NR patients (odds ratio (OR) = 2.00; 95% CI = 0.68 to 5.85; *P *= 0.2837), but it was not associated with any better neurological outcome in terms of CPC score in patients presenting with SR rather than NR (OR = 0.87; 95% CI = 0.28 to 2.68; *P *= 1.0000). Rather, the odds for good neurological outcome were more in favour of NR (pulseless electrical activity/asystole).

## Conclusion

Although TMH might be associated with better survival chances in patients presenting with SR, neurological outcome was no better (rather worse) in this group of patients when compared with patients with NR as the first documented rhythm.

